# The benefits of rehabilitation exercise in improving chronic traumatic encephalopathy: recent advances and future perspectives

**DOI:** 10.1186/s10020-023-00728-0

**Published:** 2023-09-22

**Authors:** Yin-qiong Huang, Zhe Wu, Shu Lin, Xiang-rong Chen

**Affiliations:** 1 Department of Endocrinology, The Second Affiliated Hospital of Fujian Medical University, No. 34 North Zhongshan Road, Quanzhou, 362000 Fujian China; 2 Department of Neuronal Surgery, The Second Affiliated Hospital of Fujian Medical University, No. 34 North Zhongshan Road, Quanzhou, 362000 Fujian China; 3 Centre of Neurological and Metabolic Research, The Second Affiliated Hospital of Fujian Medical University, No. 34 North Zhongshan Road, Quanzhou, 362000 Fujian China; 4 Group of Neuroendocrinology, Garvan Institute of Medical Research, 384 Victoria St, Sydney, Australia

**Keywords:** Traumatic encephalopathy syndrome, Chronic traumatic encephalopathy, Rehabilitation exercise, Neuropathology, Nerve regeneration

## Abstract

Traumatic encephalopathy syndrome (TES) is used to describe the clinical manifestations of chronic traumatic encephalopathy (CTE). However, effective treatment and prevention strategies are lacking. Increasing evidence has shown that rehabilitation training could prevent cognitive decline, enhance brain plasticity, and effectively improve neurological function in neurodegenerative diseases. Therefore, the mechanisms involved in the effects of rehabilitation exercise therapy on the prognosis of CTE are worth exploring. The aim of this article is to review the pathogenesis of CTE and provide a potential clinical intervention strategy for CTE.

## Introduction

In recent years, the concept of traumatic encephalopathy syndrome (TES) has been proposed to describe the clinical manifestations of CTE. TES is a progressive process in which patients present with cognitive dysfunction and neurobehavioral disorders (Katz et al. 2021). However, the current understanding of TES remains still not completely clear, and there is a lack of effective preventive and therapeutic strategies.

Exercise has been shown to be beneficial in the prognosis of a variety of diseases (Dibben et al. 2021; Kanaley et al. 2022). The pathophysiology of CTE may be related to abnormal protein accumulation, neuroinflammation, microcirculation injury. Studies have demonstrated that rehabilitation exercise can reduce abnormal protein deposition by enhancing signal pathway transduction, promote neurogenesis, promote synaptogenesis, and increase synaptic plasticity (Xu et al. 2022a; Horowitz et al. 2020; Mu et al. 2022). Neuroinflammation is a common pathological manifestation of a variety of nervous system diseases, including neurodegenerative diseases and central nervous system tumors, and rehabilitation exercises also help to reduce neuroinflammation (Miguel et al. 2021a; Pang et al. 2022a). Several studies have demonstrated the effectiveness of rehabilitation exercise in the treatment of neurodegenerative diseases (Ruiz-González et al. 2021a; López-Ortiz et al. 2021a; Johansson et al. 2022), and rehabilitation exercise, as a less expensive and convenient treatment, may also have a good effect on improving the prognosis of patients with CTE, so it is necessary to investigate this area. This article discusses some of the identified and possible molecular mechanisms of CTE pathogenesis, and explores the potential of rehabilitation exercise to improve the clinical manifestations of TES.

## Clinical phenotype and possible molecular mechanisms of traumatic encephalopathy syndrome

CTE has a unique pathological profile, and its diagnosis can currently only be confirmed by neuropathological examination at autopsy (Murray et al. 2022a). The specific molecular mechanism of CTE pathogenesis is not fully understood, and there are few studies on CTE from the perspective of neurovascular units (NVU), so some of the identified and possible pathogenesis of CTE are listed below.

### Pathological and clinical features of CTE

Although the consensus defines CTE-related clinical syndromes as TES, the diagnosis of TES does not represent the diagnosis of CTE, and the diagnosis of CTE still requires neuropathological examination (Katz et al. 2021). In addition, the existence of TES remains difficult to determine, and the subjective judgment of the physician is sometimes required in the TES diagnostic process, which can introduce bias (Cullum and LoBue 2021). Patients with CTE are mostly veterans, contact sports athletes, and civilians who have suffered long-term head violence in various situations (Mez et al. 2021). At present, the etiology of CTE is not clear, and it may be highly related to repetitive head blows, and a small number of patients have no clear history of neurotrauma (McKee et al. 2023). McKee divided CTE into four stages according to the severity of p-tau lesions. With progression through the stages, the patients' clinical symptoms gradually worsen (McKee et al. 2013). The first stage mainly manifests as cognitive impairment and headache; psychiatric features (usually depression and suicide) persist in the second to fourth stages; the third stage usually shows behavior disorder; dementia, motor problems and severe mental disorders are common in the fourth stage (McKee et al. 2013). It should be noted that the relationship between pathological changes and clinical symptoms has not been confirmed (Iverson et al. 2019).

### Molecular mechanisms of CTE

The gross pathology can show ventricular dilation and brain atrophy, usually located in the frontal and temporal lobes (McKee et al. 2015). The pathological changes of CTE mainly involve abnormal protein deposition, neuroinflammation, and microcirculation disorders, among which the typical microscopic pathological features are the aggregation of abnormal P-tau protein in the neurons and astroglia distributed around vessels in the deep cerebral cortical groove (McKee et al. 2016; Kaufman et al. 2021). The misfolded tau protein can induce the template misfolding and aggregation of healthy tau molecules in healthy cells, spread in a prion like manner, and then develop the lesions to other regions of the brain (Brunello et al. 2020; Falcon et al. 2019). P-tau lesions most often affect five regions of the brain: the dorsolateral frontal cortex, the superior temporal cortex, the entorhinal cortex, the amygdala, and the locus coeruleus (Alosco et al. 2020, 2019). In addition, diffuse amyloid β (A β) plaque and pathological inclusion bodies composed of transactive response DNA-binding protein can be seen in some CTE cases (Smith et al. 2019). Aβ deposition may result from axonal damage and loss, resulting in increased release of amyloid precursor protein (APP) (Chaves et al. 2021; Ikonomovic et al. 2017).

Microglia are the main cells mediating inflammation in various diseases of the central nervous system (Pang et al. 2022b; Khan et al. 2023). The activation of microglia in CTE may create conditions for maintaining chronic neuroinflammation and promote the accumulation of P-tau (Verboon et al. 2021). In addition, abnormal protein deposition can cause neuroinflammation, and RHIs can also directly cause neuroinflammation, such as the activation of Toll-like receptor myeloid difference factor 88 (MYD88) after RHIs, the promotion of nuclear factor kappa B (NF kappa B) transcription, and an increase in the expression of nucleotide-binding oligomerization domain-like receptor family pyrin domain-containing-3 (NLRP3), interleukin 1 (IL-1), and interleukin 18 (IL-18) (Bauernfeind et al. 2009). Elevated inflammatory cytokines, such as tumor necrosis factor- α (TNF- α) and interleukin-4, can induce an increase in arginase activity and expression (Thornhill and Haskard 1990). Arginase has an inhibitory effect on nitric oxide synthase (eNOS), which can reduce the production of eNOS-derived nitric oxide (NO), resulting in vasodilation dysfunction that affects parenchymal perfusion (Shin et al. 2019; Mahdi et al. 2020). In addition, neuropathological examination of CTE patients reveals disruption of the blood–brain barrier, which may be associated with loss of tight junction complex 5 (claudin-5) (Farrell et al. 2019). This may lead to a vicious circle of neuroinflammation, microcirculatory disorder and neuronal death.

**Fig. 1:**
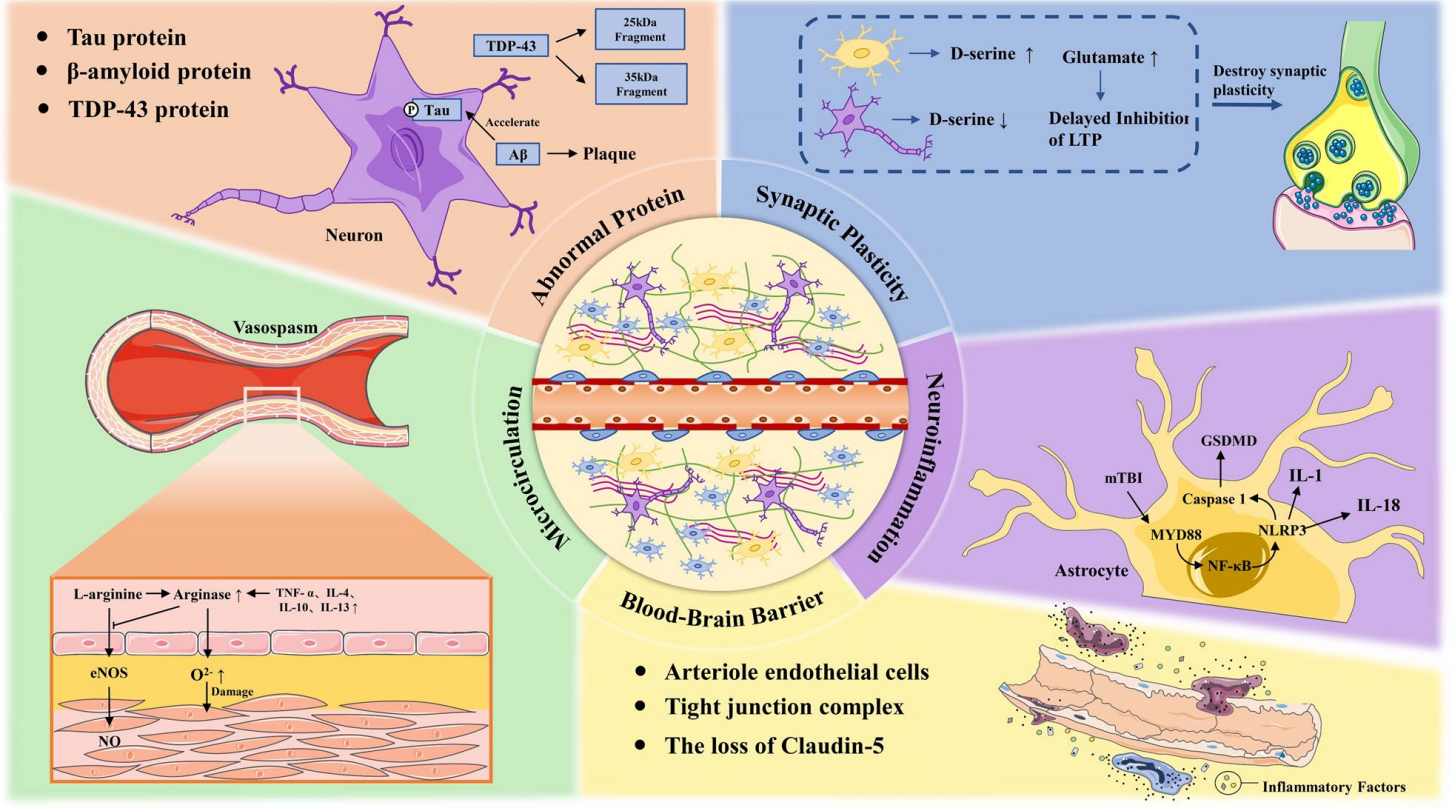
The pathological mechanisms of CTE. The accumulation of abnormal protein deposition, reduction in synaptic plasticity, and abnormal activation of the inflammatory reaction, reduced microcirculation function, and impaired blood–brain barrier are the main pathological features of CTE. It worsens the brain microenvironment and leads to extensive neuronal degeneration

Inflammation and microcirculation damage can reduce synaptic plasticity. The mechanism may be related to up-regulation of D-serine level by astrocytes, and an increase in excitatory neurotransmitter glutamate levels (Wolosker et al. 2016; Tapanes et al. 2022). Therefore, how to inhibit neuroinflammation, microcirculation disorder and neuron death, promote neurogenesis and increase synaptic plasticity are the keys to improving the prognosis of CTE (Fig. 1).

## Potential mechanisms by which rehabilitation exercises improve CTE

Rehabilitation exercise can improve cognitive function, clinical symptoms such as depression and anxiety (Pearce et al. 2022a), and can also protect the nervous system by inducing the production of brain-derived neurotrophic factor (BDNF), insulin-like growth factor I and vascular endothelial growth factor (VEGF), which has a beneficial effect on brain plasticity (Ruiz-González et al. 2021a; Morland et al. 2017a). For example, light and moderate aerobic treadmill exercise can reduce fatigue, improve neonatal hippocampal neuron survival, and improve neurobehavioral outcomes after neurological disease (Karelina et al. 2021). Regular physical exercise has been shown to be an effective way to slow the progression of neurodegenerative diseases such as Alzheimer’s disease (Pearce et al. 2022a).

**Fig. 2:**
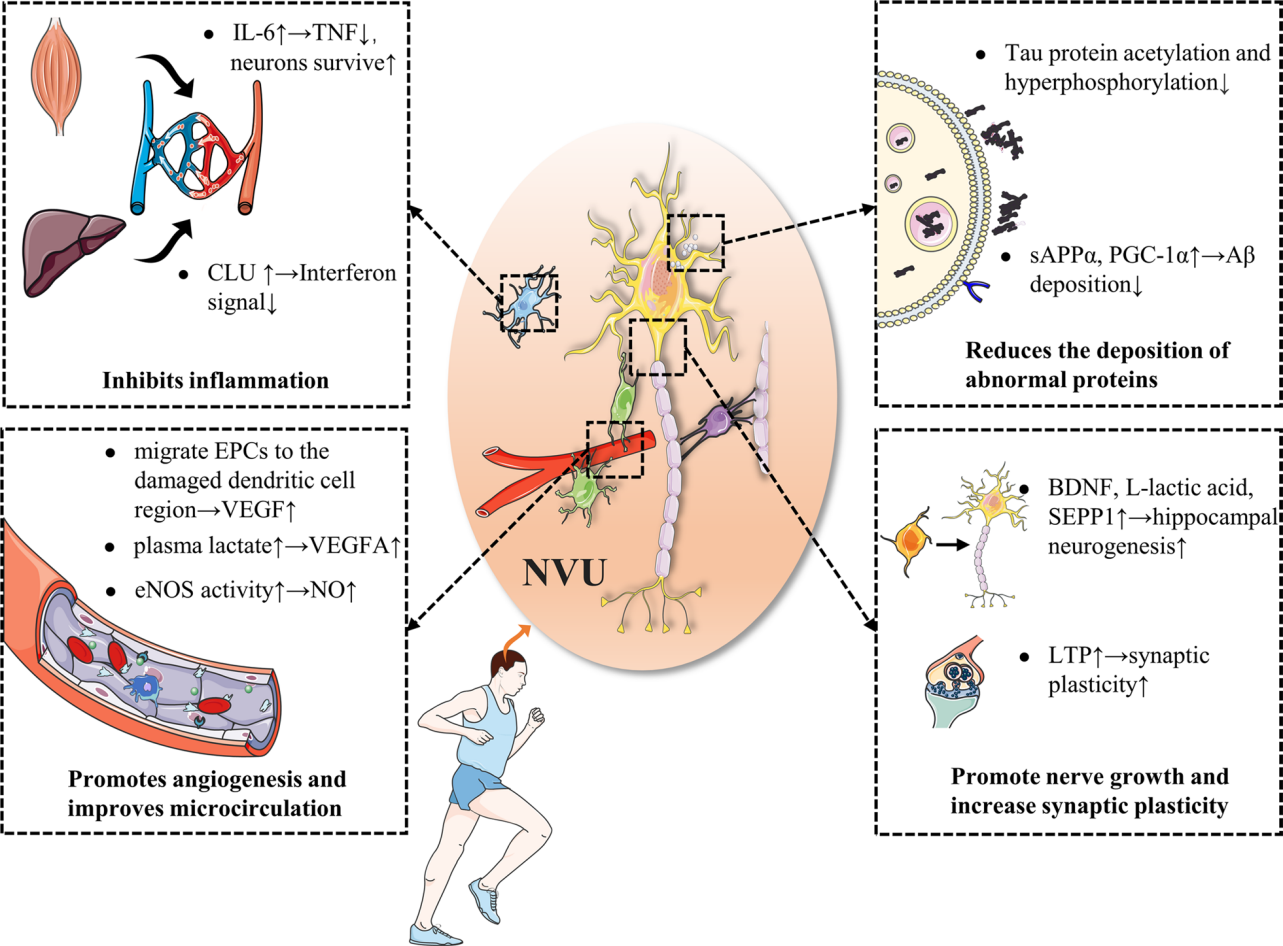
The mechanisms of exercise in CTE. CTE triggers neurodegenerative changes. However, rehabilitation exercise reduces the pathological processes of CTE through a variety of mechanisms (such as reducing abnormal protein accumulation, promoting neurogenesis, synaptic formation, increasing synaptic plasticity and promoting angiogenesis, improving microcirculation, and resisting micro-inflammation)

Repeated concussions can be a trigger for CTE (McKee et al. 2023), and rehabilitative physical activity for post-concussion patients has been shown to improve symptoms and speed recovery (Howell et al. 2021; Leddy et al. 2021). CTE progresses slowly, and as a neurodegenerative disease, rehabilitation exercises may also be effective for it (Murray et al. 2022b). In this section, we aim to introduce the potential mechanism of rehabilitative physical exercise in improving CTE. It may be related to preventing abnormal protein deposition, alleviating neuroinflammation, regulating microcirculation, and promoting neurogenesis and synaptic plasticity (Table 1) (Fig. 2).

**Table 1:** The main beneficial mechanisms of rehabilitation exercise for CTE

Sorting	Subclass	Mechanism	References
Reduction of abnormal protein deposition	Tau protein	Rehabilitation exercises can reduce tau protein acetylation and promote Wnt/GSK3 β and PI3K/Akt signaling pathways, reducing P-Tau production and increasing its clearance	Mankhong et al. (2020); Chen et al. (2020); Xu et al. (2022b)
	β-amyloid protein	Rehabilitation exercise increases ADAM10 expression and promote PGC-1 α / FNDC5 pathway thereby reduces Aβ production	Yu et al. (2021a); McMeekin et al. (2020)
Antiinflammation and oxidation stres	IL-6, CLU	Rehabilitation exercise promotes the release of IL-6 from skeletal muscles and the release of CLUs from the liver to reduce neuroinflammation	Chow et al. (2022); Bateman et al. (2016)
Promote angiogenesis and improve micro-circulation	VEGF	Rehabilitation exercise can increase plasma lactic acid to promote ERK1/2 and Akt signal transduction and promote EPC to secretion VEGF	Morland et al. (2017b); Ross et al. (2014)
Promotion of neurogenesis	BDNF	BDNF promotes neuronal development and differentiation through the BDNF/TrkB signaling pathway	Colucci-D’Amato et al. (2020)
	L-lactic acid	L-lactate activates HCA1 to promote the AKT/PK pathway to promote cell survival and value-added	Lambertus et al. (2021)
	MCT2	Rehabilitation exercise increases MCT2 expression and improves neuronal energy metabolism	Lev-Vachnish et al. (2019); Yu et al. (2021b)
The promotion of synaptogenesis and the increase of synaptic plasticity	LTP	Regular rehabilitation exercises can increase LTP	Vivar and Praag (2017)
	Glutamate	Rehabilitation exercise increases the excitatory neurotransmitter glutamate	Andersen et al. (2021); Maddock et al. (2016)

### Rehabilitation exercises reduce abnormal protein deposition

The deposition of p-tau is an important pathological feature of CTE. Deposition of other proteins such as Aβ can accelerate p-tau deposition, aggravating pathological outcomes. However, rehabilitation exercise can reduce the deposition of abnormal proteins and thus could benefit those with CTE.

#### Rehabilitation exercises reduce P-tau generation

Rehabilitation exercises can reduce P-tau synthesis and increase P-tau clearance. Tau protein acetylation can promote Tau protein aggregation and hyperphosphorylation, and make P-tau more difficult to degrade, while regular aerobic rehabilitation exercise can reduce Tau protein acetylation and improve the pathological process of CTE (Mankhong et al. 2020). In addition, rehabilitation exercise can inhibit tau protein hyperphosphorylation. Glycogen synthase kinase-3 (GSK-3) is involved in the regulation of many key cell biological pathways, and one of its subforms, GSK-3β, is involved in phosphorylation of Tau protein after CTE (Hernandez et al. 2013; Moszczynski et al. 2018). Rehabilitation exercises reduce GSK-3β activity and reduce Tau phosphorylation by inhibiting the expression of Dickkopf-1, an inhibitor of the Wnt/GSK3β pathway, by upregulating the Wnt/GSK3β pathway (Chen et al. 2020). Finally, rehabilitation can activate the PI3K/Akt pathway to increase the expression of its downstream protein HSP70, a key protein that makes up the ubiquitin–proteasome system, so rehabilitation can enhance this system and promote P-tau clearance (Xu et al. 2022b).

#### Rehabilitation exercise reduces Aβ deposition

Amyloid precursor protein (APP) is an important protein for the growth and development of neurons and the implementation of various activities (Liu et al. xxxx). APP processing pathways are generally divided into the amyloid pathway (mediated by secretary enzyme β and γ) and non-amyloid (mainly mediated by secretary enzyme α) (Cho et al. 2022; Wilkins and Swerdlow 2017). Research on the APP processing pathway shows that exercise can increase the secretion of a disintegrin and metalloprotease 10 (ADAM10), the main member of the α secretary enzyme, which increases the expression of soluble APP by increasing the non-amyloid pathway α (sAPP α) (Yu et al. 2021a). In addition, the increase in sAPPα can inhibit BACE1, thereby reducing the amyloid pathway and Aβ production (Nigam et al. 2017). Rehabilitation exercise can also reduce Aβ-induced neurological dysfunction by increasing muscle expression of proliferator-activated receptor-γ coactivator-1α (PGC-1α) and through the PGC-1α/FNDC5/BDNF pathway (Azimi et al. 2018; Neto et al. 2023). Generally speaking, rehabilitation exercises can reduce abnormal proteins (P-tau and Aβ), therefore rehabilitation exercises may be used as a potential clinical intervention strategy for the treatment of CTE.

### Rehabilitation exercise inhibits the vicious cycle of neuroinflammation—microcirculation disorders—neuronal death

Neuroinflammation is the initiating factor of secondary brain injury, which can lead to microcirculation disorders. Brain tissue and neuronal ischemia and hypoxia aggravate inflammatory reaction, forming a vicious cycle of neuroinflammation-microcirculation disorders-neuronal death. Many studies have shown that rehabilitation exercise could effectively inhibit this vicious circle, thereby improving the prognosis of patients.

#### Rehabilitation exercise has an anti-inflammatory effect

The mechanism of rehabilitation exercise to reduce neuroinflammation may be related to reducing microglia activation and promoting the release of anti-inflammatory factors from skeletal muscle and the liver. Glia mediates neuroimmunity, and intervention in glial cells through different pathways may alter disease progression (Goenka et al. 2023; Xuan et al. 2022). For example, microglia-targeted immunotherapy has good promise in tumor treatment (Pang et al. 2022a). Similarly, rehabilitation exercise helps to regulate microglia metabolic status, reduce microglia activation, and thus reduce neuroinflammation. IL-6 released from skeletal muscle after exercise can enter the brain through the blood brain barrier. IL-6 demonstrates a dual effect of promoting inflammation and exerting anti-inflammatory actions at various levels (Forcina et al. 2022), on the one hand, a transient increase in IL-6 after exercise can combine with other anti-inflammatory factors to inhibit inflammation such as inhibiting the pro-inflammatory effect of TNF (Chow et al. 2022; Bateman et al. 2016), on the other hand, long-term high systemic levels of IL-6 have pro-inflammatory effects and are associated with the occurrence of a variety of diseases, however, exercise individuals have lower baseline levels of IL-6 (Pedersen and Febbraio 2012), and functional neurogenesis refilling of post-CTE microglia may also be mediated by interleukin 6 (IL-6) trans-signaling pathways (Willis et al. 2020). IL-6 may be the key to enhancing the survival of neurons after CTE. In addition, some studies have shown that rehabilitation exercise can promote clusterin (CLU) production in the liver, targets brain endothelial cells, inhibits interferon signals, and plays an anti-inflammatory role (Miguel et al. 2021b).

#### Promotes angiogenesis and improves microcirculation

Rehabilitation exercise with a certain intensity can improve cerebral blood flow perfusion (Steventon et al. 2020). As a heparin binding growth factor, VEGF can target vascular endothelial cells, promote their proliferation and induce angiogenesis in vivo (Ahmad and Nawaz 2022). The increase in VEGF caused by exercise may be achieved through the following two ways: (1) Exercise increases the plasma lactate concentration, activates ERK1/2 and Akt signals by acting on the lactate receptor, thereby increasing the expression of vascular endothelial growth factor A (VEGFA) (Morland et al. 2017b); (2) rehabilitation exercise can promote the migration of endothelial progenitor cells (EPCs) to the area of injured dendothelial cells, and the secretion of VEGF to promote vascular growth and repair (Ross et al. 2014). In addition, rehabilitation exercise can promote EPCs to secrete EPC-derived exosomes and promote microvascular regeneration, thereby improving microcirculation (Wang et al. 2020; Ma et al. 2018). Rehabilitation exercises can also help improve vascular function. Exercise can increase vascular laminar shear stress, which can induce increased calcium-mediated eNOS enzyme activity in vascular endothelial cells, thereby increasing NO production (Tryfonos et al. 2022). Therefore, rehabilitation exercise is conducive to avoiding vascular endothelial dysfunction and reducing vascular oxidative damage. To sum up, rehabilitation exercise may induce angiogenesis, and VEGF may improve microcirculation disorders and nerve function, which may be a potential therapeutic target for improving microcirculation disorders in CTE patients.

### Rehabilitation exercises can promote nerve growth and increase synaptic plasticity

The brain is structurally and functionally highly plastic, and central nervous system diseases can have harmful effects through this property, such as neurodegenerative diseases, where the hippocampus is remodeled and cognitive and functional impairment can be caused (Weerasinghe-Mudiyanselage et al. 2022). Physical exercise has been shown to promote beneficial brain remodeling and improve neural function by regulating epigenetic inheritance and promoting the expression of neurotrophic factors (Liang et al. 2021). The positive effects of rehabilitation exercise on the brain plasticity of CTE patients are mainly reflected in promoting hippocampal neurogenesis and increasing synaptic plasticity.

#### Rehabilitation exercises contribute to hippocampal neurogenesis

During the pathological development of CTE, degenerative necrosis of neurons often occurs (Bauernfeind et al. 2009). However, the adult hippocampus can produce a type of neuron (dentate gyrus granule cells) that can eventually differentiate into new neurons (Zhou et al. 2022). Rehabilitation exercises can improve the hippocampal environment and promote adult hippocampal neurogenesis through various mechanisms, thereby improving the pathological outcome of CTE and the prognosis of patients. Rehabilitation exercise raises the level of BDNF, and its mechanism may be related to the Wnt/ β-catenin signal pathway (Cheng et al. 2020). Rehabilitation exercise can change the morphology of astrocytes and promote the expression of BDNF (Li et al. 2021). BDNF is an important neurodynamic factor in the brain, which promotes the development and differentiation of neurons and plays an active role in the repair of nerve injury (Walsh and Tschakovsky 2018). Its role in adult hippocampal neurogenesis is mediated by BDNF/TrkB signaling pathway (Colucci-D'Amato et al. 2020).

Rehabilitation exercise may promote neurogenesis by mediating L-lactic acid, and its mechanism may be associated with the hydroxycarboxylic acid receptor 1 (HCA1)-mediated AKt/PK pathway (Lambertus et al. 2021). Also, L-lactic acid can activate monocarboxylate transporter 2 (MCT2) on newborn neurons (Lev-Vachnish et al. 2019). However, overexpression of MCT2 can increase mitochondrial biogenesis, thereby improving neuronal energy metabolism (Yu et al. 2021b). Rehabilitation training can also promote hippocampal neurogenesis in adults by altering blood composition. Selenium is an indispensable trace element in the human body. The secreting selenoprotein P (SEPP1) metabolized in the liver is the organic form of selenium in the human body, which has antioxidant and anti-inflammatory effects (Hariharan and Dharmaraj 2020; Burk and Hill 2015). Studies have shown that rehabilitation exercise can significantly increase the concentration of SEPP1 in plasma and promote the binding of SEPP1 to the receptor. Researchers have reported that low-density lipoprotein receptor related protein 8 promoted hippocampal neurogenesis (Leiter et al. 2022). In addition, rehabilitation exercise can increase the concentration of glycosylphosphatidylinositol (GPI)-specific phospholipase D1 in plasma, and alter the signal cascade downstream of GPI anchor substrate lysis, thereby affecting neurogenesis, improving age-related regeneration and reducing cognitive impairment (Leiter et al. 2022; Fujihara and Ikawa 2016). Finally, rehabilitation training can also induce platelet activation, promote the secretion of platelet factor 4, and contribute to the neurogenesis of the dentate gyrus of the hippocampus (Leiter et al. 2019).

#### Rehabilitation exercises help to increase synaptic plasticity

Synaptic plasticity is an important component of learning and memory (Magee and Grienberger 2020). The progress of CTE is usually accompanied by the loss of synaptic connections, the rupture of neuronal axes and the loss of dendritic spines, which seriously damage the plasticity of synapses. In recent years, more and more data have shown that hippocampal synaptic plasticity can be achieved through regular rehabilitation exercise. Regular rehabilitation exercises can increase long-term enhancement (LTP), a form of synaptic plasticity and an important component of memory formation and maintenance (Vivar and Praag 2017). The mechanism of rehabilitation exercise-enhanced LTP is complex and may be related to a variety of exercise-induced products (Vints et al. 2022). Rehabilitation exercise can also remotely regulate synaptic plasticity through exosomes, studies have shown that rehabilitation exercise can promote exosome release and promote synaptic remodeling through microglia (Li et al. 2022; Jiang et al. 2023). Finally, rehabilitation exercises also affect the expression of neurotransmitters, thereby altering synaptic plasticity, especially levels of glutamate, which is thought to be the main excitatory neurotransmitter involved in synaptic formation and synaptic communication (Andersen et al. 2021; Maddock et al. 2016). Therefore, rehabilitation exercise may promote hippocampal neurogenesis and synaptic plasticity in a variety of ways to improve the prognosis of CTE.

At present, there are still few clinical studies and basic studies on rehabilitation exercise to improve CTE, and many patients with CTE are athletes and soldiers, compared with other patients such as civilians who have suffered from long-term head violence, they have performed long-term regular exercise before CTE, which will have an impact on the prognosis of CTE is unknown, so the specific molecular mechanism of rehabilitation exercise to improve CTE still needs to be explored and verified.

## Clinical therapeutic intervention strategy of rehabilitation exercise for CTE

**Fig. 3:**
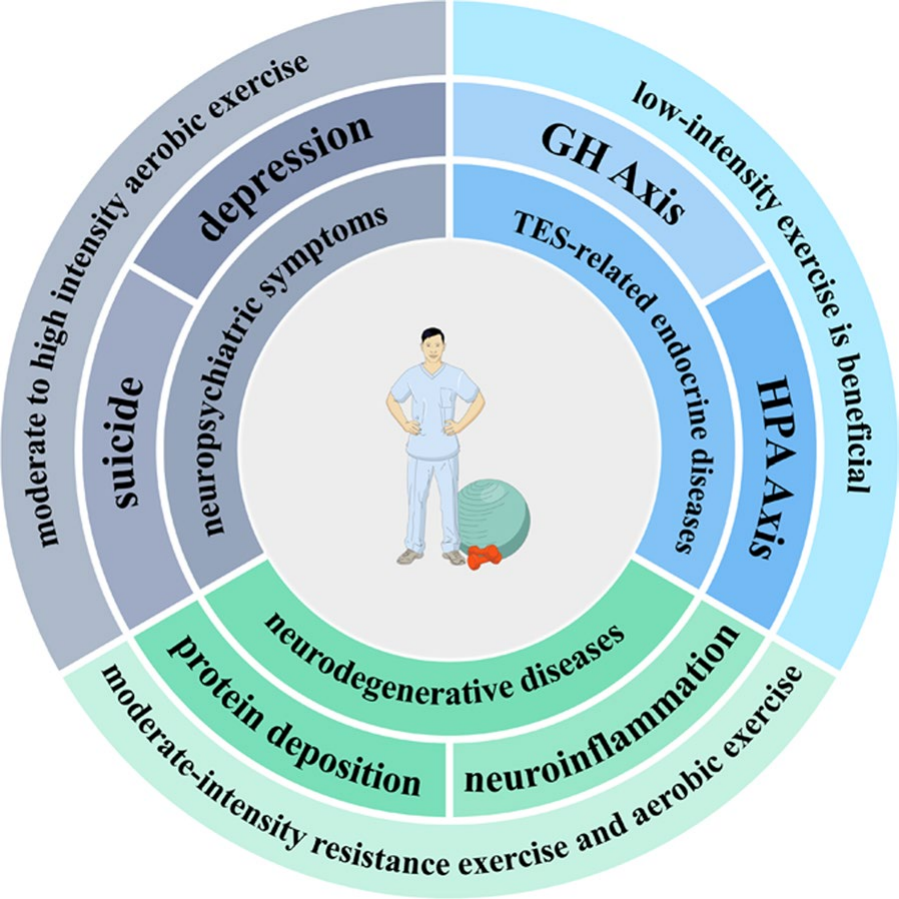
Rehabilitation exercise has obvious clinical benefits for CTE patients, which can effectively improve the symptoms of CTE-related endocrine diseases, and can significantly improve early neuropsychiatric symptoms and late diseases that are difficult to effectively treat with drugs such as AD

As the pathogenic factors and pathological mechanism of CTE have not been fully defined, and the diagnosis of its corresponding clinical syndrome TES is also challenging, the treatment of CTE is still in an emerging field. P-tau accumulation is a key pathological mechanism of CTE, so blocking P-tau deposition is also regarded as a promising target for the treatment of CTE (Kim et al. 2023). There have been studies to counter the accumulation of P-Tau by introducing adeno-associated virus vectors encoding anti-P-Tau antibodies to the central nervous system (Sacramento et al. 2020), and there have been studies using nanocapsules to deliver immunoglobulins to the central nervous system, which also help reduce the accumulation of P-tau (Zhang et al. 2023). Although these studies have shown good effects, they are still confined to animal experiments, and the effectiveness of treatment for CTE patients may still need to be verified for a long time. At the present stage, rehabilitation exercise may still be one of the most potential and easiest to implement CTE treatment. Rehabilitation exercise also has a good therapeutic effect on the complications of CTE, including related metabolic disorders such as hypopituitarism, neurobehavioral disorders, cognitive dysfunction, dementia and other adverse consequences (Fig. 3).

### Rehabilitation exercises may improve CTE-related endocrine diseases

Pituitary dysfunction is a common concomitant endocrine complication after CTE, which can lead to the loss of pituitary hormones in patients, including growth hormone, adrenocorticotropic hormone, and thyroid-stimulating hormone deficiency (Costanza et al. 2011). For these patients, different intensities and different types of exercise have different effects on patients lacking different kinds of hormones. Studies have shown that high intensity exercise is effective in increasing growth-hormone axis (GH Axis) activity, while the effect of resistance exercise on hypothalamic–pituitary–adrenal axis (HPA Axis) has not been confirmed (Haunhorst et al. 2022), but moderate and high-intensity aerobic exercise seems to increase HPA Axis activity (Hill et al. 2008; Takahashi et al. 2022). The effect of exercise on hypothalamic-pituitary-thyroid axis is uncertain (Babić Leko et al. 2021). The above evidence suggests that rehabilitation exercise may improve endocrine diseases related to CTE.

### Rehabilitation exercises may improve neuropsychiatric symptoms caused by CTE

The early manifestations of CTE in patients are neuropsychiatric symptoms, mainly depression and suicide. The effect of rehabilitation exercise on neuropsychiatric symptoms is positive (Kim 2022), and close in magnitude to that of traditional drug treatment (Smith and Merwin 2021). The mechanism of rehabilitation exercise to improve neuropsychiatric symptoms may be related to the change in neural plasticity (Smith and Merwin 2021). Rehabilitation exercise has a good effect on patients of different ages (Philippot et al. 2022; Hidalgo and Sotos 2021). Even low-intensity exercise has obvious benefits for improving neuropsychiatric symptoms (Pearce et al. 2022b). Therefore, it is recommended that TES patients start rehabilitation exercise as soon as possible.

### Rehabilitation exercise may improve neurodegenerative diseases caused by CTE

Dementia is the terminal manifestation of CTE, which is similar to the clinical presentations of many neurodegenerative diseases. CTE is also considered to be associated with neurodegenerative diseases, especially Alzheimer’s disease (AD) (Gardner and Yaffe 2015; Yu et al. 2020). CTE and AD have similar main neuropathological characteristics: P-tau protein has different structures but similar immunophenotypes. Aβ deposition can also be seen in some patients with TES (Falcon et al. 2019; Johnson et al. 2020). The clinical characteristics of CTE and AD are similar, which often leads to misdiagnosis. The effect of rehabilitation exercise on Alzheimer’s disease has been well established (Valenzuela et al. 2020). It can decrease abnormal protein deposition (Tan et al. 2021), alleviate neuroinflammation and oxidative stress (Maddock et al. 2016; Miguel et al. 2021b), improve microcirculation (López-Ortiz et al. 2021b), reduce neuronal apoptosis (Zhao et al. 2021), promote neural repair (Ruiz-González et al. 2021b), and improve the prognosis of Alzheimer’s disease caused by CTE. The recommended modality for AD is at least 45 min of moderate-intensity resistance exercise and aerobic exercise (Northey et al. 2018).

It is important to note that different types of rehabilitation exercise, such as aerobic exercise or resistance exercise, as well as the time, intensity and frequency of rehabilitation exercise, often have different effects on the mitigation of neurological diseases. Therefore, follow-up studies are needed to determine the most effective rehabilitation exercise treatment strategy for CTE. In addition, it is necessary to develop personalized strategies for patients with different behavioral abilities in the clinical implementation process to facilitate the smooth development of treatment. For some patients with obvious cognitive impairment, cognitive rehabilitation may be more conducive to symptom relief and progress. At present, there are still few studies on drug development targeting the pathological mechanism of CTE, most of which focus on immunotherapy to slow down P-tau deposition. The therapeutic targets of other pathogenic pathways remain to be discovered, and the therapeutic effects of rehabilitation therapy for CTE, including rehabilitation exercise, still need to be further verified although they have good prospects.

## Conclusion

The clinical syndrome of CTE, TES, begins early in injury and is difficult to relieve with existing means. This article reviews the potential mechanism of physical rehabilitation exercise in the treatment of CTE from various aspects, aiming to explore the prospect of rehabilitation exercise, an effective and easy-to-implement treatment, for the application of CTE.

Further research into the neuropathology of CTE patients is needed, as it can provide valuable information for the development of disease biomarkers and the evaluation of potential treatments. In addition, the impact of rehabilitation exercises on brain health in CTE patients that needs to be validated is important for improving our understanding of the mechanisms of neurodegenerative diseases, and the search for the most appropriate exercise strategy for CTE patients should continue, which may be a promising area of research in the future.

## Data Availability

All relevant data is contained within the article. Further inquiries can be directed to the corresponding author.

## Abbreviations

CTE: Chronic traumatic encephalopathy
P-Tau: Hyperphosphorylated tau protein
TES: Traumatic encephalopathy syndrome
Aβ: Amyloid-β
APP: Amyloid precursor protein
BACE1: B-site APP cleaving enzyme 1
MYD88: Myeloid difference factor 88
NF kappa B: Nuclear factor kappa B
NLRP3: Nucleotide-binding oligomerization domain-like receptor family pyrin domain-containing-3
IL-1: Interleukin 1
IL-18: Interleukin 18
TNF-α: Tumor necrosis factor-alpha
NO: Nitric oxide
eNOS: Nitric oxide synthase
ET-1: Endothelin-1
MMPs: Matrix metalloproteinases
NFAT: Nuclear cell membrane proteases
Claudin-5: Complex 5
BDNF: Brain-derived neurotrophic factor
VEGF: Vascular endothelial growth factor
GSK-3: Glycogen synthase kinase-3
ADAM10: A disintegrin and metalloprotease 10
sAPPα: Souble APP α
PGC-1α: Proliferator to activate receptor gamma coactivator-1α
IL-6: Interleukin 6
CLU: Clusterin
SOD: Superoxide dismutase
VEGFA: Vascular endothelial growth factor A
EPCs: Endothelial progenitor cells
MCT2: Monocarboxylate transporter 2
SEPP1: Selenoprotein P
GPI: Glycosylphosphatidylinositol
PDE-4: Phosphodiesterase-4
cAMP: Cyclic adenosine monophosphate
LTP: Long-term potentiation
GH Axis: Increasing growth-hormone axis
HPA Axis: Hypothalamic–pituitary–adrenal axis
AD: Alzheimer’s disease
